# ComHub: Community predictions of hubs in gene regulatory networks

**DOI:** 10.1186/s12859-021-03987-y

**Published:** 2021-02-09

**Authors:** Julia Åkesson, Zelmina Lubovac-Pilav, Rasmus Magnusson, Mika Gustafsson

**Affiliations:** 1 Department of Physics, Chemistry and Biology, Linköping University, 581 83 Linköping, Sweden; 2 Systems Biology Research Centre, School of bioscience, University of Skövde, 541 28 Skövde, Sweden

**Keywords:** Gene regulatory networks, Hubs, Master regulators, Network inference

## Abstract

**Background:**

Hub transcription factors, regulating many target genes in gene regulatory networks (GRNs), play important roles as disease regulators and potential drug targets. However, while numerous methods have been developed to predict individual regulator-gene interactions from gene expression data, few methods focus on inferring these hubs.

**Results:**

We have developed ComHub, a tool to predict hubs in GRNs. ComHub makes a community prediction of hubs by averaging over predictions by a compendium of network inference methods. Benchmarking ComHub against the DREAM5 challenge data and two independent gene expression datasets showed a robust performance of ComHub over all datasets.

**Conclusions:**

In contrast to other evaluated methods, ComHub consistently scored among the top performing methods on data from different sources. Lastly, we implemented ComHub to work with both predefined networks and to perform stand-alone network inference, which will make the method generally applicable.

## Background

The status of living organisms can to a great extent be revealed by studying its transcriptome and the underlying gene regulatory system [1]. The advent of big data techniques, such as microarray profiling and later RNA-Seq, has made data of such systems widely available to the research community for deeper analysis [2]. As a result of long-standing efforts to make sense of big biological data, we now have a corresponding plethora of methods to reverse engineer gene regulatory networks (GRNs) [3–10]. These methods are often applied to make predictions on biological properties, such as screening for drug targets [11], identifying biomarkers for diagnosis, and make prognoses of complex diseases.

However, GRN research is still facing profound challenges, as seen in previous studies showing great need for improving most available methods [12]. These poor performances ultimately hinder the progression in research on complex diseases. Due to the low accuracy of predictions the usability of GRNs has been limited, since individual gene-gene predictions cannot be trusted with any reasonable certainty [13]. The poor accuracy is well exemplified by the outcome of the DREAM5 challenge, which was a broad effort to measure the performance of GRN inference methods [12]. In the DREAM5 challenge, participants were given gene expression data from biological and *in silico*-generated networks, with instructions to predict gene-to-gene interactions. Among all predictions for the biologically derived networks, no participants achieved a higher accuracy than 35% among the top 100,000 ranked gene-gene interaction predictions. Nevertheless, gene-gene interaction predictions are not the only feature that can be extracted from a reverse-engineered GRN.

It has been noted that methods for GRN predictions tend to have a large variance in performance between different datasets, meaning that there is no method that works best for all input data profiles [13]. Therefore, statistical entities that are based on many interactions in the inferred network, called hubs, has frequently been used for systems biology analysis [14–16]. Moreover, to the best of our knowledge no corresponding large scale assessment of methods for hub inference has yet been performed. As of today, there are only a few methods available for hub inference from expression data. One common approach is to first infer modules using weighted correlation network analysis (WGCNA) and then identify hubs based on module connectivity [17–19]. Another tool is the master regulator inference algorithm (MARINa) [20], which has been successfully used in several studies aiming to infer hubs [21]. However, MARINa is a tool that builds on already predicted GRNs [20], and is thus dependent on the often poor ability of individual GRN inference algorithms to recreate networks from expression data. In addition, MARINa is only applicable when data from two phenotypes are available. Indeed, most approaches for extracting hubs from gene expression data that are based on prior GRN inference methods vary greatly in performance between datasets. Nevertheless, the analysis of the DREAM5 network inference challenge showed that combining the predictions of methods into a crowd estimate increases accuracy and improves robustness of network inference [12]. To solve the problem of inconsistent hub-prediction performance, and to transfer the insights of community predictions of gene regulations into a hub inference context, we herein present the Community Hub prediction algorithm, ComHub.

**Fig. 1:**
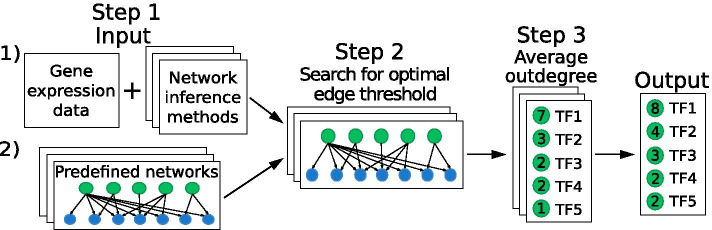
The workflow of ComHub. ComHub either takes as input (1) Gene expression data and applies a set of network inference methods or (2) predefined networks. ComHub identifies an optimal number of edges to include from the predicted GRNs. Next, the outdegrees of each transcription factor is averaged over the method predictions

ComHub is a method that uses a meta-prediction of regulator outdegree based on independent network prediction algorithms. Moreover, ComHub was inspired by the community network approach presented in the analysis of the DREAM5 challenge outcome [12]. We found, analogously, that the methods’ ability to predict hubs can be improved by combining the outdegrees of regulators into a community. ComHub works by first applying a compendium of GRN inference methods to predict gene-gene interactions. Second, ComHub computes the outdegrees of regulators for each method and combines the predictions by averaging the outdegrees (Fig. 1). We benchmarked ComHub using the datasets aquired in DREAM5 challenge, and observed it to outperform the DREAM5 community approach on biological datasets. To verify the soundness of ComHub, and to further exemplify the usefulness of hub predictions, we lastly applied ComHub to independent data from two different sources: *Bacillus subtilis* gene expression data and gene expression profiles across human tissues. We confirmed ComHub to again achieve robust predictions of hubs. We implemented ComHub as a Python package, available for download from https://gitlab.com/Gustafsson-lab/comhub.

## Results

### An intra-community assessment identifies better edge prediction thresholds

We developed ComHub by analysing how to combine predictions of the 35 network inference methods that were present in the DREAM5 challenge, where networks were reverse engineered from *E. coli* and *in silico* gene expression data. Specifically, we focused on the predicted outdegree of each regulator, and how network predictions of multiple inference methods should be combined to most accurately predict system hubs.

**Fig. 2:**
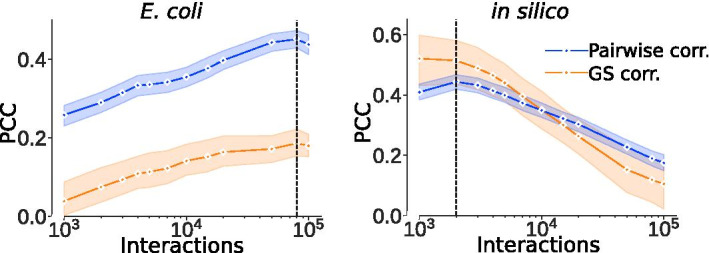
The similarity between methods coincides with the optimal performance. The average pairwise correlation, assessed with Pearson correlation coefficient (PCC), between method predictions (blue) compared to the average method performance (orange) for the *in silico* and *E. coli* datasets. The dotted lines shows were the optimal edge threshold were selected

GRN inference methods generally predict thousands of interaction predictions with varying degrees of confidence. How many of these predicted interactions that can be considered accurate is an unsolved problem of GRN inference, and we also noted that the regulator outdegree is largely influenced by the number of considered edges. We therefore first sought an independent measure to identify a threshold for edge inclusion into the ComHub algorithm. Such a measure is not obvious, but we hypothesised the similarity between ingoing network inference methods to be a good predictor of how posed the problem was. In other words, we reasoned that the meta-prediction should be the most well-defined at the threshold of included edges that also maximised the similarity of outdegree-rankings between the ingoing methods. We assessed the similarity between the GRN predictions from the DREAM5 challenge as a function of number of considered interactions, ranked by prediction confidence. As a similarity measure we used the correlation of regulator outdegree between each pair of predicted GRNs. Moreover, we compared the similarity with the average performance of the network inference methods (Fig. 2). Notably, we observed that the number of considered edges that yielded the highest correlation between methods also corresponded to the threshold of optimal performance. This observation was important as it allows for an unbiased approach to estimate the number of edges to be included in the GRN prediction. Moreover, we speculated that such measures of intra-algorithm consistency can be used to give an unbiased estimation of the information embedded in the data also across the field of GRN inference.

Using the prediction filtering from the intra-algorithm prediction correlations, we estimated the optimal number of included edges to be 80,000 and 2000 for the *E. coli* and *in silico* datasets, respectively. Correspondingly, compared to the gold standard network the true optimal edge numbers were 80,000 and 1000, with optimal performance either being exactly overlapped (*E. coli*), or within 2% of the optimal performance for *in silico*. Hence, the pairwise correlation between methods can be used as a measurement on how many inferred interactions that should be included in a community prediction.

### ComHub robustly predicts hub transcription factors on the DREAM5 challenge data

**Fig. 3:**
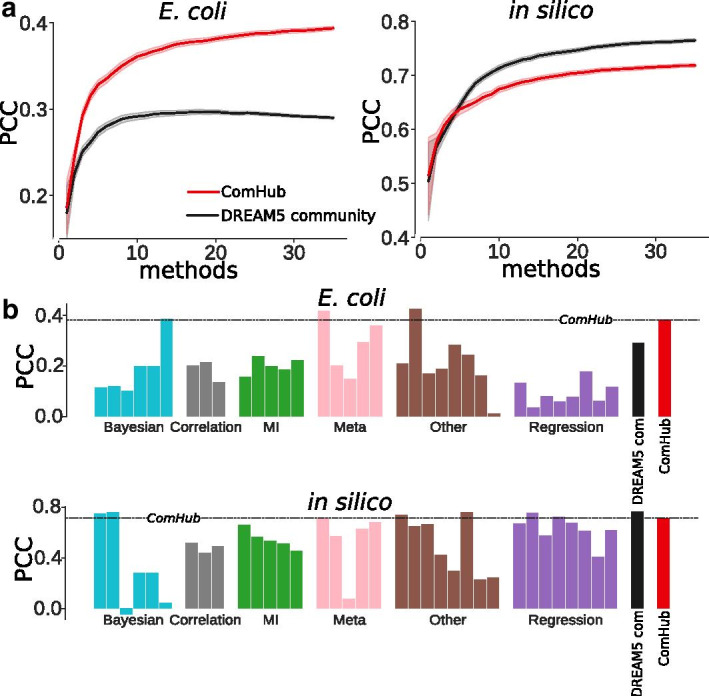
The performance of ComHub on the in silico and E. coli datasets. **a** The performance of ComHub (red) and the DREAM5 community network (black) as a function of in-going methods. **b** The maximal performance (combining 35 method predictions) of ComHub, the DREAM5 community network approach and each of the DREAM5 participant methods, on the *in silico* and *E. coli* datasets

As noted by Marbach et al. [12], GRN inference methods have an overall diverse performance depending on data properties. This variation also applies to the ability to predict hubs, as measured by gene outdegree. To address this limitation, we applied ComHub to predict hubs based on the combined results of the DREAM5 participants’ inferred networks, and measured performance as the Pearson correlation coefficient (PCC) between outdegree in the prediction and the gold standard network. Furthermore, the performance was also evaluated using absolute error and HITS hub score [22] shown in Additional file 1: figures 1 and 2. By applying ComHub to sets of randomly drawn networks from the DREAM5 contestants, we observed a robust network, converging with the gold standard as the number of in-going methods increased (Fig. 3a). Notably, this convergence occured at a relatively low number of in-going DREAM5 predictions, and at only six methods the average PCC measured 85% and 90% of the maximal PCC for *E. coli* and *in silico*, respectively. When including all 35 methods, we observed a PCC between the predicted and gold standard outdegree of 0.38 and 0.71 for *E. coli* and *in silico*, respectively (Fig. 3b). In addition, we compared the performance of ComHub to the community approach presented in the DREAM5 challenge, where the consensus was taken on each gene-gene interaction individually. ComHub outperformed the community approach from the DREAM5 challenge on *E. coli* (Fig. 3b).

### ComHub was validated on *B. subtilis* and human gene expression data

We verified the applicability of ComHub using two independent datasets. First, we used *B. subtilis* gene expression data along with the gold standard from [23]. Second, we used a compendium of human gene expression data across tissues and cell lines from the Human Protein Atlas [24], along with the human protein-protein interaction network from STRINGdb as a gold standard [25]. We applied 6 GRN inference methods, covering a variety of different approaches. These methods included a bootstrap ElasticNet (bootstrap=1000) [3], the “trustful inference of gene regulation with stability selection” (TIGRESS) [4], the “context likelihood of relatedness” (CLR) [5], the “algorithm for the reconstruction of accurate cellular networks” (ARACNE) [6], the absolute value of the PCC [7], and the “gene network inference with ensemble of trees” (GENIE3) [8]. The computing efficiency of each of these methods are shown in Additional file 1: Supplementary Fig. 3.

**Fig. 4:**
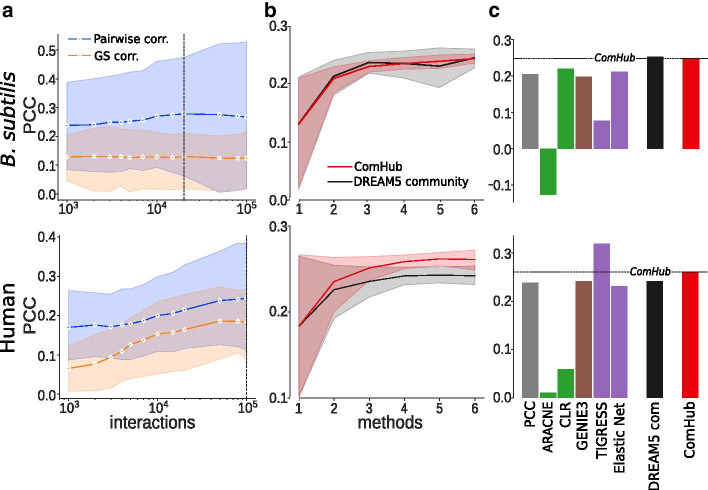
The performance of ComHub on the B. subtilis and human gene expression datasets. **a** The pairwise correlation between the method predictions (blue), compared to average method performance (orange). The dotted lines shows where the edge thresholds were selected. **b** The performance of ComHub (red) and the DREAM5 community network approach (black) as a function of in-going methods. **c** The performance of ComHub, the DREAM5 community network approach and each of the 6 network inference methods used

For the *B. subtilis* dataset, ComHub identified the number of interactions to be used in the predicted network to 50,000 by assessing the similarity between the methods’ predictions, as described above (Fig. 4a). This point coincided with a smaller peak in performance for individual methods. For the human dataset, the peak in similarity at 100,000 interaction clearly coincided with the peak in performance for individual methods. For both datasets, the greatest improvement of performance occurs already with only a few in-going methods to ComHub, with the PCC to saturate already at approximately four included GRN inference methods (Fig. 4b). ComHub placed among the the top performing methods on both datasets; clearly outperforming the individual methods on the *B. subtilis* dataset and almost all individual methods on the human dataset (Fig. 4c). The performance of ComHub compared to the DREAM5 community of individual edges was similar on the *B. subtilis* dataset while ComHub performs better on the human dataset.

### ComHub compared to the WGCNA

**Fig. 5:**
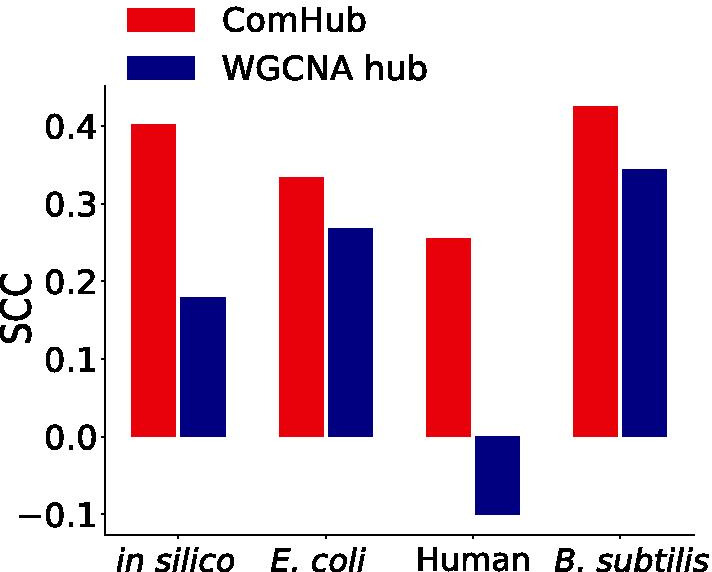
The performance of ComHub (red) compared to the WGCNA hub approach (blue). The performance was assessed using Spearman correlation coefficient (SCC). Interestingly, ComHub outperformed WGCNA in all comparisons, and in particular succeeded on the human gene expression data, whereas WGCNA even resulted in a negative correlation between predicted hub rankings and the gold standard

ComHub is a method that predicts master regulators, i.e. hubs, from the gene regulatory networks from independent GRN inference algorithms. However, there are alternative methods for inferring hubs from gene expression data. Therefore, we lastly aimed to compare ComHub to such hub methods. Several studies have identified hubs by first applying WGCNA for module detection and then using a connectivity measure of each gene to identify hub genes [17–19]. We applied the WGCNA approach on the four datasets studied herein, resulting in lists of regulators ranked on the regulators module connectivity. To compare the performance of ComHub with the WGCNA approach, we evaluated each approach towards the regulator outdegree in the gold standards using the Spearman correlation coefficient. We found ComHub to outperform the WGCNA approach on all four datasets (Fig. 5). In addition, a gene set enrichment analysis of the hub predictions on human data can be found in Additional file 1: Figure 4. As expected, the hub predictions are enriched for Gene Ontology terms related to the general function of transcription factors, with many terms shared between the different methods predictions. ComHub had enrichment for more terms than WGCNA hub and many of the GRN inference methods predictions.

## Discussion

The importance of deriving biological network properties as key factors in regulatory actions has gained a lot of attention, especially in the emerging field of network medicine. Yet, the role of hubs as crucial nodes in GRNs is still to be explored further. Moreover, the absence of gold standard methods for hub prediction triggers the need for benchmarking existing approaches and developing novel improved approaches. Herein we present a novel hub prediction approach, ComHub, which improves hub predictions by integrating GRN predictions from a variety of standard gene-gene interaction identification approaches.

Several benchmark studies have previously been performed to evaluate network inference methods’ ability to predict several aspects of GRNs [12, 26, 27]. The DREAM5 challenge, being one of the biggest GRN inference algorithm benchmarks with its 35 methods included, assessed the accuracy of predicted interactions. Another benchmark performed by [26] instead assessed network inference methods’ ability to preserve topology and complexity of GRNs. These benchmarks all draw the conclusion that network inference methods show a generally poor performance with no method being the clear winner in all settings. The poor performance of network inference methods on data from different sources was also observed for predicting hubs in GRNs. There is no universal inference method that will perform well on data from all sources. This varying performance between GRN inference methods makes choosing one method suitable for a specific dataset a difficult task. To solve this problem we developed ComHub, which by combining multiple methods produces estimations of degree. ComHub was evaluated on data from four different data sources, continuously producing hub predictions among the top performing methods.

A potential limitation of ComHub, compared to the evaluated network inference methods, is that the regulatory strength of regulator-target gene interactions should not be explicitly interpreted. To infer hubs as well as regulator-target gene interactions other network inference methods would be necessary to use. As of today, methods that predict gene-gene interactions are abundant, although the DREAM5 comparison pinpoints that their performance to predict specific interactions vary greatly across different datasets. Few methods, however, focus on the importance of gene regulators, i.e. hubs. We designed ComHub to combine individual interaction predictions of independent edge-inference methods, and benchmarked ComHub by comparing the results with outdegree rankings of the DREAM5 methods. Moreover, we note that the primary focus of DREAM5 did not include hub predictions. Nevertheless, summating the outdegrees in a predicted gene regulatory network remains one of the most common means to also predict hubs. Degree is contrary to global topological measures as betweenness centrality a local measure of importance that is robust to false individual interaction inferences. Therefore, our intention was to show that this property could be inferred robustly with higher accuracy than edge predictions. The main reason being that it is an aggregated sum over many individual edge predictions, which by the central limit theorem has lower expected relative variance than the average individual predictions.

We built ComHub with the hypothesis that the combination of several GRN inference methods improves hub predictions. Combining methods to produce community predictions has successfully been applied to GRN inference, with one example being the community approach from the DREAM5 challenge. In here, we tested a similar strategy for the inference of genes with high outdegrees, i.e. hubs. As hubs have been found to have a particular impact in systems biology, we believe the inference of such to be a significant advancement within systems biology of differentiation studies [14, 28] and systems medicine central to complex diseases and cancers [15, 16].

We discovered that the greatest improvement in performance occurs when combining only a few methods, with no significant gain in performance made when combining more than approximately six methods. Hence, ComHub can improve hub inference from as little as six GRN inference methods, which makes ComHub a relatively calculation efficient tool to use. Furthermore, the number of interactions used for hub predictions is often chosen by setting an arbitrary threshold [14]. We observed that this threshold largely affects the performance of the predictions and can differ widely depending on the dataset. ComHub utilizes a data-driven approach to in an unbiased way select how many interactions the GRN predictions should contain to maximise the accuracy of hub predictions. The need for a data-driven approach was clearly shown by the big difference in optimal threshold for the *in silico* and *E. coli* networks.

We implemented ComHub as a Python package, aiming to provide an easy-to-use method for hub predictions. ComHub includes the possibility to run six independent network inference methods using the same formatted input data. In addition, the user has the option to incorporate in-house network inference methods, providing a flexibility which we believe will broaden the use of ComHub. Herein, we focused on transcription factors as the main regulators of gene expression. Nevertheless, ComHub can take an arbitrary set of genes to use as explanatory variables, and in extension, rank by node outdegree. Future work will also include integrating ComHub into a user friendly web application with focus on disease biomarker discovery, to make it available for the broader research community.

## Conclusions

We developed ComHub, a tool which improves hub predictions by combining the predictions of several GRN inference methods. ComHub is built as a Python package, with six well-used GRN inference methods incorporated and the option to include in-house network inference methods. A data-driven approach is applied to select how many edges to include from the GRN predictions, before averaging the regulator outdegrees. ComHub continuously produced robust hub predictions when benchmarked on data from the DREAM5 challenge as well as two independent datasets. Furthermore, ComHub offers possible applications in hub-based biomarker discovery, which could benefit the field of network medicine. For example, ComHub could be used to discover disease regulators important for the prognoses and treatment of complex diseases.

## Methods

### ComHub workflow

Herein we present ComHub, a novel tool for predicting network hubs from reverse engineered GRNs. ComHub makes hub predictions by averaging regulator outdegrees over a compendium of reverse engineered GRNs (Fig. 1). ComHub operates in three steps. First, if no a priori putative networks exist, ComHub takes gene expression data and a list of potential gene expression regulators as input, e.g. transcription factors. ComHub applies a set of GRN inference methods. The GRN inference methods outputs predicted regulator-target interactions, ranked based on a method specific confidence score. Second, a threshold on the number of predicted edges to include from the GRN predictions is set. ComHub includes the number of top ranked edges that maximises the correlation between the regulator outdegrees of the GRN predictions. Last, ComHub calculates the average outdegree of each regulator according to:

$$\begin{aligned} score_k=\frac{1}{N} \sum _{l=1}^{N} outdegree_{kl} , k \in \{1,R\}, l \in \{1,N\}, \end{aligned}$$

where N is the number of GRN predictions and R is the number of regulators. The output of ComHub is a list of regulators ranked on the average outdegree ($score_k$).

We implemented ComHub as a Python 3 package which utilizes widely used GRN inference methods. To capture properties across all types of approaches, we sough to include default methods of a broad set of inference classes. From the field of linear regression, we implemented a bootstrap ElasticNet (bootstrap $=$ 1000) [3], and the “trustful inference of gene regulation with stability selection” (TIGRESS) [4]. Mutual information methods added were the “context likelihood of relatedness” (CLR) [5] and the “algorithm for the reconstruction of accurate cellular networks” (ARACNE) [6]. We also incorporated the correlation-based method of calculating the absolute value of the PCC [7], and the tree-based GRN inference method “gene network inference with ensemble of trees” (GENIE3) [8]. To not limit ComHub to the default methods, we also added the option for the user to directly input GRNs of any chosen inference method.

### Benchmarking ComHub on the DREAM5 challenge

We benchmarked ComHub on predictions of 35 network inference methods from the DREAM5 challenge, where networks were reverse engineered from *E. coli*, and *in silico* gene expression data. The DREAM5 challenge included methods covering a variety of approaches such as regression, mutual information, correlation, Bayesian and meta predictors. In the DREAM5 challenge, each of the 35 participants delivered a maximum of 100,000 ranked inferred interactions between transcription factors and target genes. We also compared ComHub to the outdegree rankings that resulted from the community approach developed by [12] that is based on integrating predictions across inference methods by averaging over the ranked edges. The performances of the methods were evaluated with the gold standard networks that were accompanied with the DREAM5 challenge. Furthermore, the performances were defined as the PCC between regulator outdegree in the GRN predictions and the gold standard.

### Benchmarking ComHub on independent data

We applied ComHub to two independent gene expression datasets; *B. subtilis* gene expression data [23], and a compendium of gene expression data across tissues and cell lines from the Human Protein Atlas [24]. The *B. subtilis* gene expression data consisted of microarray data from two studies (GSE67023, GSE27219). Known transcription factors were obtained from the database of transcriptional regulation in *B. subtilis* (DBTBS) [29] and a gold standard were obtained from [23]. The Human Protein Atlas data consisted of RNA-Seq data from 37 tissues and 64 cell lines. For the human dataset we used a list of possible transcription factors from the DBD: Transcription factor prediction database [30]. As a gold standard we used a protein-protein interaction network obtained from STRINGdb version 10.5 [25], where edges with a $\hbox {confidence score} > 700$ were included.

### ComHub compared to hub predictions of the WGCNA

We compared the performance of ComHub to a hub detection approach based on WGCNA [31]. First, an adjacency matrix was constructed from the gene expression data, adapting the soft-thresholding power for each dataset, and transformed into a topological overlap matrix. Second, the genes were divided into modules by performing hierarchical clustering, cutting the tree using dynamic tree cut, and lastly merging modules with similar expression. Third, the module connectivity of each transcription factor was calculated, by first calculating the module eigengenes and then correlating the gene expression profiles with the module eigengenes. Lastly, the transcription factors were ranked based on module connectivity, where transcription factors with a high connectivity is considered as hubs. The performance was evaluated towards the transcription factor outdegrees in the gold standard for respective dataset. The performance of the WGCNA approach were compared to the performance of ComHub using Spearman correlation coefficient.

## Supplementary Information

**Additional file 1**. Supplementary material.

## Data Availability

The software is available at https://gitlab.com/Gustafsson-lab/comhub. All data analysed during this study are cited within this published article

## Abbreviations

GRN: Gene regulatory network
WGCNA: Weighted correlation network analysis
MARINa: Master regulator inference algorithm
PCC: Pearson correlation coefficient
TIGRESS: Trustful inference of gene regulation with stability selection
CLR: Context likelihood of relatedness
ARACNE: Algorithm for the reconstruction of accurate cellular networks
GENIE3: Gene network inference with ensemble of trees
SCC: Spearman correlation coefficient

## References

[1] Santolini M, Barabási A-L (2018). Predicting perturbation patterns from the topology of biological networks. Proc Natl Acad Sci.

[2] Wang Z, Gerstein M, Snyder M (2009). RNA-Seq: a revolutionary tool for transcriptomics. Nat Rev Genet.

[3] Zou H, Hastie T (2005). Regularization and variable selection via the elastic-net. J R Stat Soc.

[4] Haury AC, Mordelet F, Vera-Licona P, Vert JP (2012). TIGRESS: trustful inference of gene regulation using stability selection. BMC Syst Biol.

[5] Faith JJ, Hayete B, Thaden JT, Mogno I, Wierzbowski J, Cottarel G, Kasif S, Collins JJ, Gardner TS (2007). Large-scale mapping and validation of Escherichia coli transcriptional regulation from a compendium of expression profiles. PLoS Biol.

[6] Margolin AA, Nemenman I, Basso K, Wiggins C, Stolovitzky G, Favera RD, Califano A (2006). ARACNE: an algorithm for the reconstruction of gene regulatory networks in a mammalian cellular context. BMC Bioinform.

[7] Butte AJ, Kohane IS (2000). Mutual information relevance networks: functional genomic clustering using pairwise entropy measurements. Pac Symp Biocomput.

[8] Huynh-Thu VA, Irrthum A, Wehenkel L, Geurts P (2010). Inferring regulatory networks from expression data using tree-based methods. PLoS ONE.

[9] Hernaez M, Blatti C, Gevaert O, Berger B (2020). Comparison of single and module-based methods for modeling gene regulatory networks. Bioinformatics.

[10] Logsdon BA, Gentles AJ, Miller CP, Blau CA, Becker PS, Lee SI (2015). Sparse expression bases in cancer reveal tumor drivers. Nucleic Acids Res.

[11] Aloraini A, ElSawy KM (2018). Potential breast anticancer drug targets revealed by differential gene regulatory network analysis and molecular docking: Neoadjuvant docetaxel drug as a case study. Cancer Inform.

[12] Marbach D, Costello JC, Küffner R, Vega NM, Prill RJ, Camacho DM, Allison KR, Kellis M, Collins JJ, Aderhold A, Stolovitzky G, Bonneau R, Chen Y, Cordero F, Crane M, Dondelinger F, Drton M, Esposito R, Foygel R, De La Fuente A, Gertheiss J, Geurts P, Greenfield A, Grzegorczyk M, Haury AC, Holmes B, Hothorn T, Husmeier D, Huynh-Thu VA, Irrthum A, Karlebach G, Lèbre S, De Leo V, Madar A, Mani S, Mordelet F, Ostrer H, Ouyang Z, Pandya R, Petri T, Pinna A, Poultney CS, Rezny S, Ruskin HJ, Saeys Y, Shamir R, Sîrbu A, Song M, Soranzo N, Statnikov A, Vega N, Vera-Licona P, Vert JP, Visconti A, Wang H, Wehenkel L, Windhager L, Zhang Y, Zimmer R (2012). Wisdom of crowds for robust gene network inference. Nat Methods.

[13] Magnusson R, Gustafsson M (2020). LiPLike: towards gene regulatory network predictions of high certainty. Bioinformatics (Oxford, England).

[14] Gustafsson M, Gawel DR, Alfredsson L, Baranzini S, Bjorkander J, Blomgran R, Hellberg S, Eklund D, Ernerudh J, Kockum I, Konstantinell A, Lahesmaa R, Lentini A, Robert H, Liljenstrom I, Mattson L, Matussek A, Mellergard J, Mendez M, Olsson T, Pujana MA, Rasool O, Serra-Musach J, Stenmarker M, Tripathi S, Viitala M, Wang H, Zhang H, E. Nestor C, Benson M (2015). A validated gene regulatory network and GWAS identifies early regulators of T cell-associated diseases. Sci Transl Med.

[15] Alvarez MJ, Subramaniam PS, Tang LH, Grunn A, Aburi M, Rieckhof G, Komissarova EV, Hagan EA, Bodei L, Clemons PA, Dela Cruz FS, Dhall D, Diolaiti D, Fraker DA, Ghavami A, Kaemmerer D, Karan C, Kidd M, Kim KM, Kim HC, Kunju LP, Langel Ü, Li Z, Lee J, Li H, Livolsi V, Pfragner R, Rainey AR, Realubit RB, Remotti H, Regberg J, Roses R, Rustgi A, Sepulveda AR, Serra S, Shi C, Yuan X, Barberis M, Bergamaschi R, Chinnaiyan AM, Detre T, Ezzat S, Frilling A, Hommann M, Jaeger D, Kim MK, Knudsen BS, Kung AL, Leahy E, Metz DC, Milsom JW, Park YS, Reidy-Lagunes D, Schreiber S, Washington K, Wiedenmann B, Modlin I, Califano A (2018). A precision oncology approach to the pharmacological targeting of mechanistic dependencies in neuroendocrine tumors. Nat Genet.

[16] Jörnsten R, Abenius T, Kling T, Schmidt L, Johansson E, Nordling TEM, Nordlander B, Sander C, Gennemark P, Funa K, Nilsson B, Lindahl L, Nelander S (2011). Network modeling of the transcriptional effects of copy number aberrations in glioblastoma. Mol Syst Biol.

[17] Niu X, Zhang J, Zhang L, Hou Y, Pu S, Chu A, Bai M, Zhang Z (2019). Weighted gene co-expression network analysis identifies critical genes in the development of heart failure after acute myocardial infarction. Front Genet.

[18] Qiu J, Du Z, Wang Y, Zhou Y, Zhang Y, Xie Y, Lv Q, Fan H (2019). Weighted gene co-expression network analysis reveals modules and hub genes associated with the development of breast cancer. Medicine (United States).

[19] Yuan L, Chen L, Qian K, Qian G, Wu CL, Wang X, Xiao Y (2017). Co-expression network analysis identified six hub genes in association with progression and prognosis in human clear cell renal cell carcinoma (ccRCC). Genom Data.

[20] Lefebvre C, Rajbhandari P, Alvarez MJ, Bandaru P, Lim WK, Sato M, Wang K, Sumazin P, Kustagi M, Bisikirska BC, Basso K, Beltrao P, Krogan N, Gautier J, Dalla-Favera R, Califano A (2010). A human B-cell interactome identifies MYB and FOXM1 as master regulators of proliferation in germinal centers. Mol Syst Biol.

[21] Wu J, Huang B, Chen H, Yin Q, Liu Y, Xiang Y, Zhang B, Liu B, Wang Q, Xia W, Li W, Li Y, Ma J, Peng X, Zheng H, Ming J, Zhang W, Zhang J, Tian G, Xu F, Chang Z, Na J, Yang X, Xie W (2016). The landscape of accessible chromatin in mammalian preimplantation embryos. Nature.

[22] Kleinberg JM (1999). Authoritative sources in a hyperlinked environment. J Am Chem Soc.

[23] Arrieta-Ortiz ML, Hafemeister C, Bate AR, Chu T, Greenfield A, Shuster B, Barry SN, Gallitto M, Liu B, Kacmarczyk T, Santoriello F, Chen J, Rodrigues CD, Sato T, Rudner DZ, Driks A, Bonneau R, Eichenberger P (2015). An experimentally supported model of the Bacillus subtilis global transcriptional regulatory network. Mol Syst Biol.

[24] Uhlen M, Fagerberg L, Hallstrom BM, Lindskog C, Oksvold P, Mardinoglu A, Sivertsson A, Kampf C, Sjostedt E, Asplund A, Olsson I, Edlund K, Lundberg E, Navani S, Szigyarto CA-K, Odeberg J, Djureinovic D, Takanen JO, Hober S, Alm T, Edqvist P-H, Berling H, Tegel H, Mulder J, Rockberg J, Nilsson P, Schwenk JM, Hamsten M, von Feilitzen K, Forsberg M, Persson L, Johansson F, Zwahlen M, von Heijne G, Nielsen J, Ponten F (2015). Tissue-based map of the human proteome. Science.

[25] Szklarczyk D, Franceschini A, Wyder S, Forslund K, Heller D, Huerta-Cepas J, Simonovic M, Roth A, Santos A, Tsafou KP, Kuhn M, Bork P, Jensen LJ, Von Mering C (2015). STRING v10: protein–protein interaction networks, integrated over the tree of life. Nucleic Acids Res.

[26] Kiani NA, Zenil H, Olczak J, Tegnér J (2016). Evaluating network inference methods in terms of their ability to preserve the topology and complexity of genetic networks. Semin Cell Dev Biol.

[27] Saelens W, Cannoodt R, Saeys Y. A comprehensive evaluation of module detection methods for gene expression data. Nat Commun. 2018;9(1). 10.1038/s41467-018-03424-4. 10.1038/s41467-018-03424-4 PMC5854612 29545622

[28] Rackham OJL, Firas J, Fang H, Oates ME, Holmes ML, Knaupp AS, Suzuki H, Nefzger CM, Daub CO, Shin JW, Petretto E, Forrest ARR, Hayashizaki Y, Polo JM, Gough J (2016). A predictive computational framework for direct reprogramming between human cell types. Nat Genet.

[29] Sierro N, Makita Y, De Hoon M, Nakai K (2008). DBTBS: a database of transcriptional regulation in Bacillus subtilis containing upstream intergenic conservation information. Nucleic Acids Res.

[30] Wilson D, Charoensawan V, Kummerfeld SK, Teichmann SA (2008). DBD - Taxonomically broad transcription factor predictions: new content and functionality. Nucleic Acids Res.

[31] Langfelder P, Horvath S. WGCNA: an R package for weighted correlation network analysis. BMC Bioinform. 2008;9(559). 10.1186/1471-2105-9-559. 10.1186/1471-2105-9-559 PMC2631488 19114008

