# Supplementary material for: ComHub: Community predictions of hubs in gene regulatory networks
Source: BMC Bioinformatics. 2021 Feb 9;22:58. doi: 10.1186/s12859-021-03987-y (PMC7871572; doi:10.1186/s12859-021-03987-y)
Supplement: Supplementary file 1 — Additional file 1. Supplementary material. [file 12859_2021_3987_MOESM1_ESM.pdf]

Supplementary material

ComHub: community predictions of hubs in gene regulatory networks

Julia Åkesson, Zelmina Lubovac-Pilav, Rasmus Magnusson, Mika Gustafsson

List of figures

Figure S1 Performance evaluated with absolute error. 1
Figure S2 Performance evaluated with HITS hub score 2
Figure S3 Computing efficiency of GRN inference methods implemented in the ComHub software. 3
Figure S4 Gene set enrichment analysis (GO terms) of hub predictions on human data. 4

Performance evaluated with absolute error

The performance of ComHub and the benchmarked gene regulatory network (GRN) inference methods was evaluated using absolute error (Figure S1). The absolute error is defined as:

$$\Delta x = |x_i - x|$$

where x_i is the inferred regulator outdegree and x is the gold standard regulator outdegree. The GRN inference methods showed an overall diverse performance, with no method outperforming the other methods when applied to data from different sources. Using absolute error as an evaluation metric, ComHub and the DREAM5 community approach showed an average performance. This was expected since both the ComHub prediction and the prediction by the DREAM5 community approach are aggregations of the predictions by the other GRN inference methods.

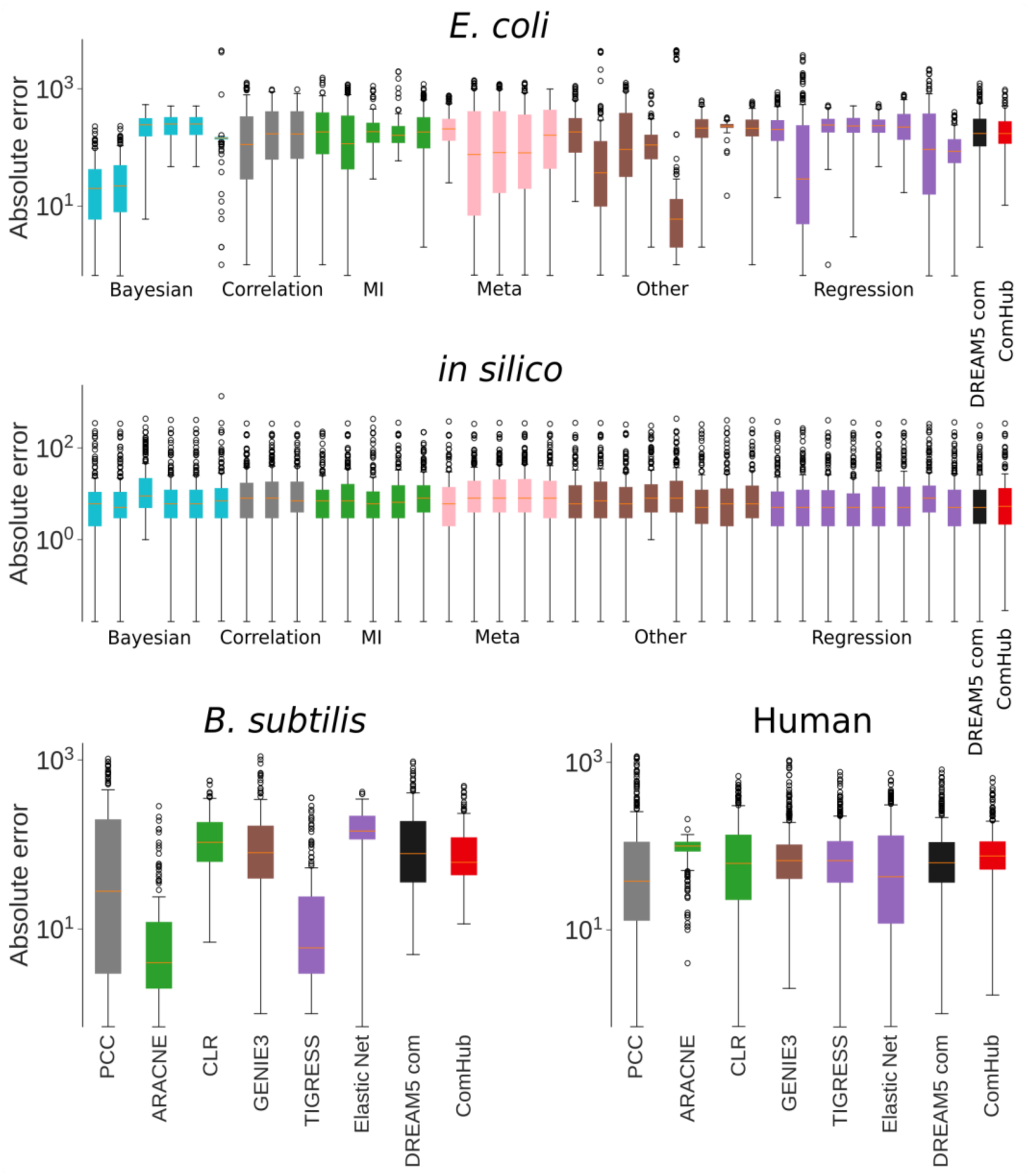

Figure S1 Performance evaluated with absolute error. The performance was assessed on the predictions by ComHub and the benchmarked GRN inference methods.

Performance evaluated with HITS hub score

The performance of ComHub, WGCNA hub and the benchmarked GRN inference methods was evaluated using HITS hub score [1] (Figure S2). First, the HITS hub score of each regulator in the gold standard was calculated using the hits() function from the Python library NetworkX [2]. Second, for each method the HITS hub scores were ordered according to each prediction ranked regulator list. Third, the Spearman correlation coefficient (SCC) was calculated between the gold standard ranked HITS hub scores and the prediction ordered HITS hub scores. The performances of the methods were similar to the performance obtained when assessing the correlation between outdegrees, with ComHub performing among the top-performing methods on all four datasets.

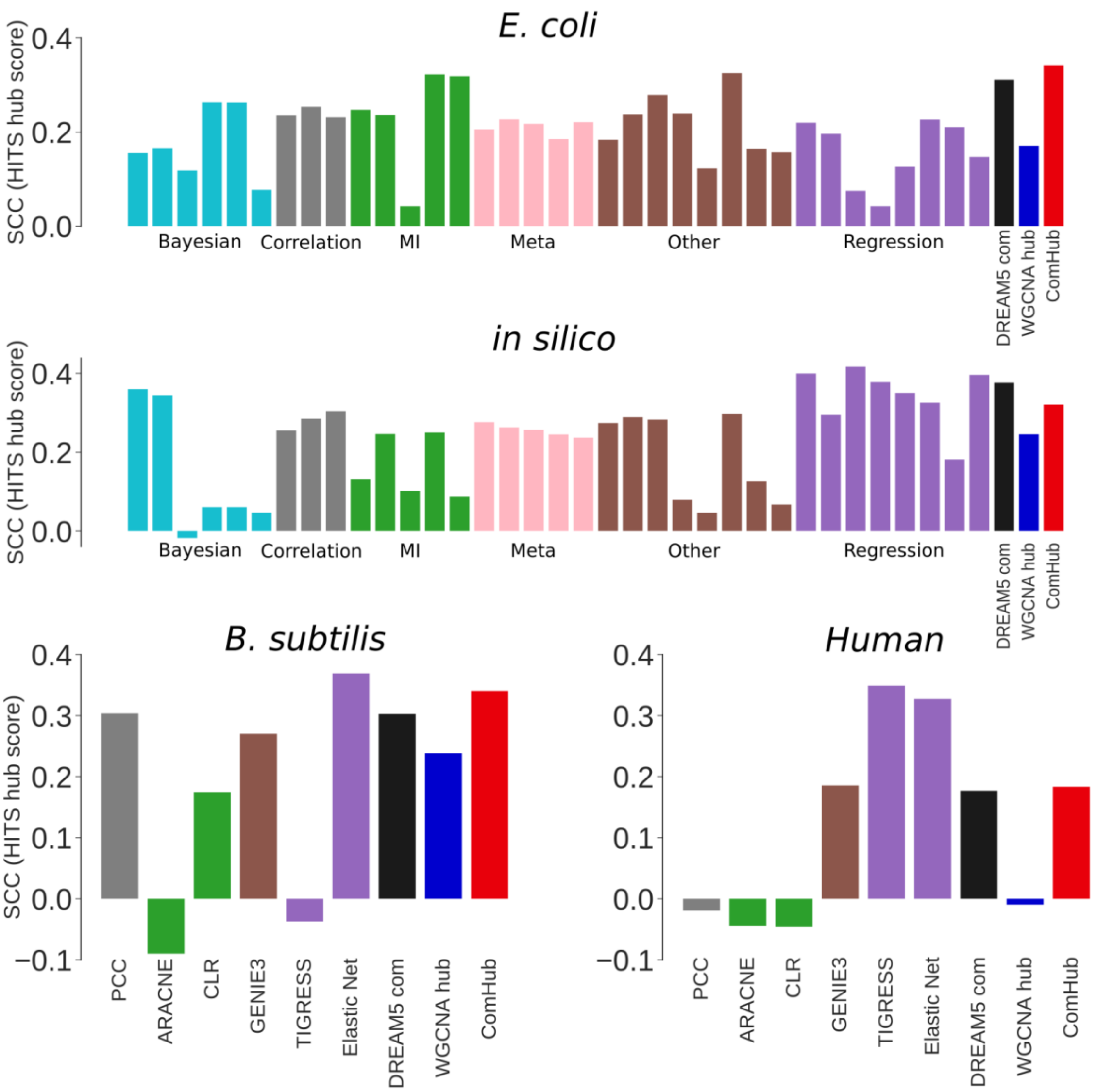

Figure S2 Performance evaluated with HITS hub score. The performance was assessed on the predictions by ComHub, WGCNA hub, and the benchmarked GRN inference methods.

Computing efficiency of GRN inference methods implemented in ComHub

The ComHub software includes six readily available GRN inference methods. These methods are by default included when ComHub is applied to expression data. However, users can opt to use a subset of these methods, and also include additional methods. The software is implemented in Python but also runs methods written in R and MATLAB. The methods CLR and TIGRESS are available both as a MATLAB-version and a R-version. The results from the performed benchmark were obtained using the MATLAB-version of the implementations. However, the R-versions of the methods are considerably faster to run (Figure S3). Figure S3 shows the computing efficiency of the GRN inference methods on the *B. subtilis* dataset, which consist of 3994 genes and 537 samples. It is important to note that the computing efficiency of Elastic Net scales linearly with the number of bootstraps. The computing efficiency was assessed on a computer with Ubuntu 18.04 and a 5-core processor.

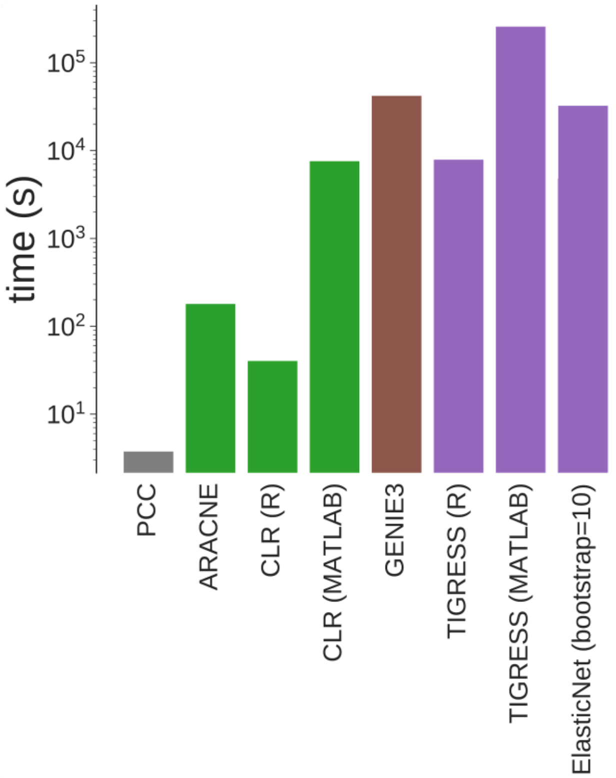

Figure S3 Computing efficiency of GRN inference methods implemented in the ComHub software. The computing efficiency was assessed on the *B. subtilis* dataset. Note that the computing efficiency of Elastic Net scales linearly with the number of bootstraps.

GSEA analysis (GO terms) of the hub-predictions on Human data

A gene set enrichment analysis for Gene Ontology (GO) terms was performed on the hub predictions on the Human Protein Atlas dataset using the R-package clusterProfiler [3] (Figure S4). For each prediction, the top-ranked 10% transcription factors (total of 92 transcription factors) were considered as hubs. Many of the enriched GO terms for the ComHub hub prediction were recurrent in several of the other methods hub predictions. This was expected since the ComHub prediction is an aggregation of the predictions by the individual GRN inference methods. The ComHub prediction was enriched for more GO terms than the WGCNA hub prediction. All the evaluated methods find hubs enriched for GO terms related to the general function of transcription factors. GO terms such as “leucine zipper domain binding”, “nuclear receptor activity”, and “histone acetyltransferase binding” only enriched in a few of the other evaluated methods were enriched in the ComHub prediction.

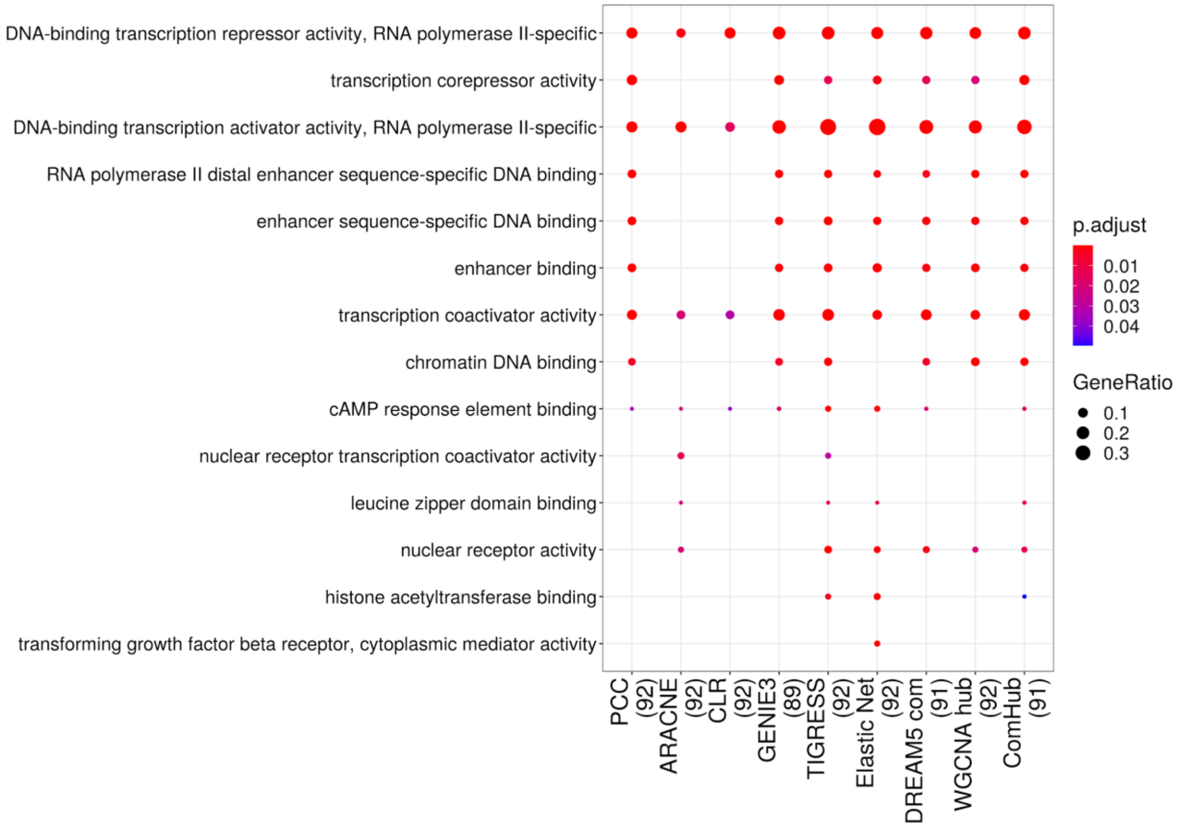

Figure S4 Gene set enrichment analysis (GO terms) of hub predictions on human data. The analysis was performed on the top-ranked 10% transcription factors in each prediction, resulting in a total of 92 hubs.

References

1. Kleinberg JM. Authoritative sources in a hyperlinked environment. J Am Chem Soc. 1999;46: 604–632. doi:10.1515/9781400841356.514
2. Hagberg AA, Schult DA, Swart PJ. Exploring network structure, dynamics, and function using NetworkX. Proc 7th Python Sci Conf (SciPy 2008), Gäel Varoquaux, Travis Vaught, and Jarrod Millman (Eds), (Pasadena, CA USA). 2008; 11–15.
3. Yu G, Wang LG, Han Y, He QY. ClusterProfiler: An R package for comparing biological themes among gene clusters. Omi A J Integr Biol. 2012;16: 284–287. doi:10.1089/omi.2011.0118
